# Medical Opinion and Movement

**Published:** 1909-10-09

**Authors:** 


### Medical Opinion and Movement.

Two cases of Torticollis which followed the removal of adenoid vegetations are reported by Weinstein in the *Medezinische Klinik*. In both cases the children had been very excited during the operation, and it had been necessary to use a considerable amount of force to keep their heads still. It is possible, therefore, that some fibres of the sterno-mastoid muscle had become overstretched or torn, resulting in the appearance of a torticollis similar to that which sometimes follows a prolonged labour with the breech presenting. Thost, however, attempts to explain such occurrences by assuming that they are the result of inflammatory changes commencing in the lymphatic ganglia. Knight, on the other hand, considers them due to injury of the filaments of the sympathetic which acts reflexly on the muscle. This explanation, nevertheless, is not confirmed by a case of Gillette's, in which tonsillectomy cured a persistent torticollis. The author himself remarks that disease of the ear, and of the tympanic cavity in particular, may cause spasm of the sterno-mastoid muscle. The prognosis in all cases is, fortunately, good.

---

An interesting case of Ligation of the Mesenteric Veins for purulent pylephlebitis following appendicitis is reported by Professor Wilms, of Basel, in the *Zentralblatt für Chirurgie*. The patient developed an appendicular abscess accompanied by rigors and fever. The abscess was opened and drained, but two days later the patient had rigors again. The temperature was of the irregular intermittent type and rigors occurred on the subsequent two days. On the supposition that the condition was an ascending pylephlebitis, the whole ileo-cæcal region was exposed by incising the peritoneum on the outer side of the cæcum and stripping intraperitoneally with the finger. The anterior aspect of the mesenteric vessels was then exposed by dividing the peritoneum overlying them. The veins were isolated from the arteries and were tied off in two small bundles. A drain was carried down to the cæcum. The patient showed no evidence of disturbed circulation in the cæcum, there were no further rigors, and convalescence was quickly established. Pylephlebitis is such a fatal complication of appendicular trouble with the treatment hitherto pursued, that this case of successful intervention reported by Professor Wilms offers a new departure in surgical procedure of considerable importance. Unfortunately no details as to the condition of the veins, etc., are given in support of the diagnosis of pylephlebitis, and the clinical course can hardly be regarded as unequivocal evidence.

General and persistent Convulsions are not a common complication of whooping-cough, but they are very likely to prove fatal. At the University Clinic of Berlin, among 476 children admitted for whooping-cough there were 176 deaths, and of these convulsive fits formed the cause of second importance with 24. In such cases post-mortem examination showed no definite cerebral lesion, but a well-marked œdema of the meninges and brain. In view of this fact, Dr. Eckert conceived the idea that this œdema might be relieved by lumbar puncture, and he has carried out the treatment in four severe cases with very satisfactory results. Some years back Dr. Bertolotti reported two cases of whooping-cough complicated with severe convulsions, in which lumbar puncture effectually relieved the condition. The four cases observed by Dr. Eckert were all infants aged 11 to 17 months. Two of the children were admitted in a profound state of coma, and in all the convulsive fits were severe and repeated; 5 to 25 c.c. of cerebro-spinal fluid were drawn off. In one case only it was necessary to repeat the operation. In the second case in which 15 c.c. were evacuated there was a pressure of 200 mm. falling immediately on lumbar puncture to 80. The lumbar puncture was always followed by a warm bath with cold douching. Dr. Eckert supposes that the œdema results from the venous stasis produced by the expiratory spasms of the cough. Lumbar puncture induces reabsorption of the œdema and the vigorous inspiratory efforts, promoted by the bath and douching, increase the circulatory activity and so help to relieve the condition.

---

Various attempts have been made in cases of General Paralysis to exercise a favourable influence on the course of the disease by the production of a condition of pyrexia by tuberculin injections or local inflammations by injections of terebinthine. On the supposition that any good effects from such treatment are the result of an induced leucocytosis, Dr. O. Fischer, of Prague, has tried the effect of injections of nucleinate of soda. He injects half-a-gramme in a 10 per cent. solution every three to five days. After each injection there was a considerable leucocytosis (16 to 25,000 per cubic millimetres) which lasted about three days. He has treated 22 cases in this way, and the course of the disease compared favourably with that of 22 other cases not so treated. Among the former there have been four deaths, while among the latter the mortality is, up to the present, eight. Professor J. Donath, of Budapest, has independently also been carrying out the same mode of treatment. He injected as much as two grammes of sodium nucleinate in 100 c.c. solution, and appears to have obtained a correspondingly intensified reaction. The average leucocytosis was 23,000, and even attained 61,000 per cubic millimetre. He gave from three to 18 injections to each patient. The results reported by Dr. Donath were much more marked than appears to have been experienced by Dr. Fischer. In 10 out of 21 cases the improvement was sufficient to enable the patients to return to work. Tremors and the condition of excitation and speech troubles disappeared; there was improvement in memory and in the ability to reckon, etc. In five other cases there was also some improvement, but in a less degree. In the remaining six cases there was no change. The first injections produce local inflammatory swellings, so that it is necessary to make them alternately on different sides of the body. According to Dr. Donath, the treatment is especially applicable to cases in which antisyphilitic measures are not indicated.

A NEW early sign of Tubercular Disease is described by Dr. Poënaro-Capleseo, of Bucharest. It consists in a swelling of the upper eyelids. According to him, it is so well marked in some patients as to strongly suggest Bright's disease; but careful examination of the urine in such cases has failed to reveal any abnormality in regard to the kidneys, and the cardio-vascular system was found to be in all respects healthy. Among 61 patients who showed this orbitopalpebral sign at a time when the tubercular disease gave no apparent indication, the author has been able to watch 23 develop, in later years, obvious signs of tuberculosis. The forms of the disease in which the sign is especially likely to present itself are tubercular glands, joints, and pulmonary tuberculosis. Some of the patients in whom the sign was well marked showed also slight symptoms of Basedow's disease: slight tachycardia, hypertrophy of the thyroid gland, so that the author suggests that this sign may be connected with an excessive activity of the thyroid gland, resulting from the noxious influences of the tubercular toxines upon the gland or upon the sympathetic or pneumogastric nerves. Although such subsidiary signs of doubtful significance cannot be relied upon in the diagnosis of disease, they may be sufficiently indicative to induce more careful examination, and they may prove of value in the unravelling of the pathological processes of a disease.

A CASE in which death resulted from the inhalation of the Concentrated Vapour of Benzine is reported by Descoeudres and Baccharach in *La Revue Médicale de la Suisse Normande*. The victim was a workman, who, after going down into a benzine-tank to draw off some of the liquid, lost consciousness almost immediately and fell to the ground muttering incoherently. His body was seized with purposeless and convulsive movements which lasted until the onset of death a few moments later. Artificial respiration was kept up some considerable time, but without effect. At the autopsy nothing abnormal was found except a few reddish-brown plaques situated in the neighbourhood of the interpleural groove, and which appeared to consist of subpleural extravasations. A blood analysis showed all the characteristic changes of asphyxia, such as the disappearance of the coagulability of the blood, a lowering of its freezing-point, and a diminution in the resisting power of the red-cells. The authors believe this case to be unique, as they have been unable to find a parallel to it in the literature.

PLEURISY, a rare complication of Scarlet Fever, is yet one which occurs with relative frequency in the course of particular epidemics. Rogery, of Paris, has recently published a study of this complication, and finds that in the majority of cases some secondary infection, such as otitis, arthritis, broncho-pneumonia, etc., and not the scarlet fever itself, is the determining factor in its evolution. It is usually at the period of desquamation that the pleurisy manifests itself, but it may appear at any stage of the disease. It may occur in the sero-fibrinous form, developing gradually without a general reaction into an abundant effusion, or it may at times be purulent from the outset, with grave constitutional disturbance, giving rise eventually to a general infection. In both varieties a microscopical examination will reveal the presence of typical chains of streptococci in the fluid. Prognosis in the sero-fibrinous form is usually good, but is much graver when the effusion is purulent. Death occurs in some 50 per cent. of these cases, despite all care. Treatment in both varieties is on the lines of that of ordinary pleurisies. If the effusion be abundant, thoracocentesis will be necessary. In the case of purulent collections a rib should be resected and thorough drainage ensured.

THE value of Inoculation as a plague preventive has frequently been demonstrated, but never so clearly as in the report of the Bombay Bacteriological Laboratory just issued. In Bangalore between October 1907 and the end of February 1909 rather more than half the population of the city was inoculated. Out of 46,986 persons so treated, 34 were attacked by plague and 13 died; among 42,613 persons not inoculated there were 1,798 cases of plague with 1,518 deaths. Major Standage reports that no ill-effects were observed as the result of inoculation. An extensive inoculation campaign was carried out in 28 villages of Kollegal Taluk, in Coimbatore district, 20,108 persons being inoculated as against 31,305 not inoculated. Among the inoculated there were 405 cases and 150 deaths. Among the uninoculated there were 1,180 cases and 921 deaths. The results are even better than the figures indicate, because many of those not inoculated were never exposed to infection. From the Azamgarh district of the United Provinces, where (according to *Indian Public Health*) an extensive inoculation campaign was carried on, it was reported that no reliable figures could be given for most of the villages, but the statistics of one village, Khalispur, are remarkable. Out of a total population of 206, 182 were inoculated in November 1908. Plague appeared in the following January, and of the 182 persons inoculated one was attacked and died, while among the 24 not inoculated there were 14 cases of plague, and 11 proved fatal. Again the advantages of inoculation as a protection for those subject to infection by the nature of their employment is strikingly illustrated by the case of Karachi. Here almost the whole of the municipal sweeper establishment was inoculated by the end of February 1908. Among 1,114 inoculated sweepers there were six cases and three deaths, while among 92 not inoculated there were 13 cases and 11 deaths. Again the results were better than the figures show, for three of the six inoculated men who suffered from plague were taken ill within five days of inoculation, and presumably had the germs of plague in them when inoculated. Many other instances could be quoted, but the figures given are sufficient to demonstrate the immense utility of inoculation as a preventive measure.

According to an article by Lafforgue in *La Province Médicale*, Constipation may frequently mask the usual symptoms of dysentery. As the result of studying two epidemics of this disease, he has been able to show that there are three varieties of constipation, which occur at different periods of the affection. The first variety, which manifests itself at the onset of the illness, is not preceded by a period of diarrhœa, so that the disease is apt to be overlooked unless a careful examination of the stools be made and the result confirmed by bacteriological examination. Treatment consists in the application of local sedative medication. The second variety is preceded by a short period of diarrhœa, and though less insidious in its onset than the preceding form, again necessitates a bacteriological examination. Treatment is the same as in the first case. The third variety occurs at the end of the course of the disease. In this constipation may be obstinate, and is apt to be mistaken by the casual observer for the end of the malady. It is due to atony of the intestine, and is accompanied by no subjective painful phenomena. If the patient be treated with laxatives and enemata, and allowed to resume his ordinary habits and diet, acute symptoms are apt to reappear. It is therefore necessary that the stools be examined carefully for Shiga's bacillus, and, if this be found, the patient must be ordered frequent purgatives and a strict diet. It is important, both from the point of view of prophylaxis and from that of the patient and his neighbours, that a complete sterilisation of the stools be carefully carried out.

---

Doyen (*Société de l'Internat*) has described a New Method of Treatment for Arterial Aneurysm in cases where it is impossible to remove the dilated vessel, as a whole, or to put it out of the general circulation by complete ligature of the vessel above the dilatation. Arguing that the aneurysm is caused by excessive arterial tension, he proposes to lessen this tension by incomplete ligature of the vessel above the dilatation. He has tried this method in the case of a popliteal aneurysm, subsequently opening up the sac of the aneurysm, turning out the clots, and after cutting away the redundant tissues of the walls, re-establishing the lumen of the vessel for a distance of some ten centimetres by a row of double sutures. The operation was completely successful. The author believes that a similar procedure would be successful in several otherwise incurable aneurysms of the large arteries of the neck and abdomen. It is possible that in certain cases this treatment might be successful, but, on the other hand, patients suffering from aneurysm are frequently subject to a more or less general arterio-sclerosis, and it would seem at least likely that a recurrence of the trouble would take place above the partial ligature unless a perfectly healthy vessel were present at the site of ligature. Until the treatment has been tried in a large number of cases it would be unwise to consider it a method that should be generally employed in the treatment of the disease.

Dr. Brecke, of Davos, has recently published the Results of his Clinical and Anatomical Researches into sixteen cases of intra-thoracic adenopathy. The symptomatology of the condition is extremely complex, but the author is of opinion that the presence of several of the following symptoms may be taken as making the diagnosis of the condition sufficiently certain. (1) The persistence of a zone of dulness with a sensation of resistence to percussion at the upper part of the sternum. (2) The presence of a systolic bruit at the same level, or to the left of this zone, in the second intercostal space. (3) Paralysis of the vocal cords. (4) Pain in the spine. (5) Gastric pain. (6) Reaction to tuberculin. Radioscopic examination is useful in confirming the diagnosis. If the disease be of some standing, a spasmodic cough and paroxysmal attacks of dyspnœa will be added to the other symptoms. As a rule it is impossible to determine whether it be the mediastinal or bronchial glands which are affected, but both groups may, of course, be simultaneously attacked. It is sometimes difficult to diagnose the condition from inflammation of the connective tissue of the mediastinum, and the possibility of similar symptoms being caused by pleuritic effusions must not be overlooked. In such cases information may be afforded by auscultation and percussion in other parts, particularly between the scapulæ, and by ascertaining whether the sympathetic be involved or not.

---

A serious complication of typhoid fever, and one which renders prognosis most grave, is the onset of Myocarditis. The most prominent symptoms of this affection are an increase in prostration of the patient, dyspnœa, and cardiac acceleration. Weakening of the beat and sounds of the heart, embryo-cardiac rhythm, cyanosis and oliguria are all proofs of cardiac paresis and herald in severe cases the approaching collapse. In the prevention of this formidable complication, Dr. Desclaux (*Journal des Praticiens de l'Ouest*) first of all pays attention to the general hygiene of the patient. Absolute rest, with the head kept low without a pillow, must be insisted upon, for the simple action of sitting up in bed has caused syncope and death. Cold baths are to be avoided as tending to collapse, but cold may be applied to the præcordia day and night by means of ice-bags constantly renewed. A piece of flannel should be placed between the bag and the skin to avoid skin lesions. This treatment should be continued for several days; it has the effect of lowering the temperature, slowing the pulse, and increasing the heart-beat. The author advocates a milk diet, and not more than three pints should be given daily to avoid hindering the action of the heart by distension of the vascular system with fluid. From 3 to 5 minims tincture digitalis should be given three times a day, and camphor, spartein, strophanthus, and strychnine sulphate should be injected alternately *sub cutem*. The author is opposed to the use of caffeine in the disease. Excitation and even delirium often supervene upon its administration, and a period of excitation may be followed by one of dangerous depression.

